# Safety and Efficacy of Sintilimab and Anlotinib as First Line Treatment for Advanced Hepatocellular Carcinoma (KEEP-G04): A Single-Arm Phase 2 Study

**DOI:** 10.3389/fonc.2022.909035

**Published:** 2022-05-31

**Authors:** Xiaofeng Chen, Wei Li, Xiaofeng Wu, Fengjiao Zhao, Deqiang Wang, Hao Wu, Yanhong Gu, Xiao Li, Xiaofeng Qian, Jun Hu, Changxian Li, Yongxiang Xia, Jianhua Rao, Xinzheng Dai, Qianwen Shao, Jie Tang, Xiangcheng Li, Yongqian Shu

**Affiliations:** ^1^Department of Oncology, Jiangsu Province Hospital, The First Affiliated Hospital, Nanjing Medical University, Nanjing, China; ^2^Department of Oncology, Pukou Branch of Jiangsu People’s Hospital, Nanjing, China; ^3^Department of Oncology, The First Affiliated Hospital, Soochow University, Suzhou, China; ^4^Hepatobiliary Center, Jiangsu Province Hospital, The First Affiliated Hospital, Nanjing Medical University, Nanjing, China; ^5^Department of Oncology, Huai’an Second People’s Hospital, The Affiliated Huai’an Hospital, Xuzhou Medical University, Huai’an, China; ^6^Department of Oncology, Affiliated Hospital of Jiangsu University, Zhenjiang, China; ^7^Department of Pathology, Jiangsu Province Hospital, The First Affiliated Hospital, Nanjing Medical University, Nanjing, China; ^8^Department of Oncology, Nanjing Red Cross Hospital, Nanjing, China; ^9^Department of Oncology, Liyang People’s Hospital, Liyang, China

**Keywords:** advanced hepatocellular carcinoma, sintilimab, anlotinib, anti-PD1, receptor tyrosine kinase

## Abstract

**Purpose:**

Immune checkpoint inhibitors plus antiangiogenic tyrosine kinase inhibitors may offer a first-line treatment for advanced hepatocellular carcinoma (HCC). In this phase 2 trial [registered with clinicaltrials.gov (NCT04052152)], we investigated the safety and efficacy of first-line anti-PD-1 antibody sintilimab plus antiangiogenic TKI anlotinib for advanced HCC.

**Methods and Materials:**

Pathologically-proven advanced HCC patients received sintilimab (200 mg) on day 1 and anlotinib (12 mg) once daily on days 1 to 14 every 3 weeks, with a safety run-in for the first six participants to assess dose-limiting toxicities (DLTs). The primary endpoints were safety and objective response rate (ORR) per RECIST v1.1.

**Results:**

Twenty advanced HCC patients were enrolled. No DLTs occurred in the safety run-in. All patients had treatment-related adverse events (TRAEs). Grade 3 TRAEs occurred in 8 (40.0%) patients, the most common being decreased platelet count (10.0%) and increased γ-glutamyl transferase (10.0%). No grade 4/5 TRAEs occurred. Five (25%) patients developed immune-related AEs. The ORR was 35.0% (95%CI 15.4%-59.2%) per RECIST v1.1 and 55.0% (95%CI 31.5%-76.9%) per modified RECIST. At data cutoff (March 31, 2021), the median progression-free survival was 12.2 months (95%CI, 3.8 to not reached). The median PFS was significantly longer in patients with lower LDH levels (not reached [NR], 95% CI, 8.7 to NR vs. higher LDH levels 5.2 months, 95% CI 3.4 to NR; *P*=0.020) and a CONUT score ≤2 (NR, 95% CI 5.1 to NR vs. CONUT score >2 6.2 months, 95% CI 1.8 to NR; *P*=0.020). Furthermore, patients showing tumor response had a significantly higher median proportion of CD16^+^CD56^+^ NK cells than patients who had stable or progressive disease (21.6% vs. 14.6%; P=0.026).

**Conclusion:**

Sintilimab plus anlotinib showed promising clinical activities with manageable toxicity as first-line treatment of advanced HCC.

## Introduction

Primary liver cancer is the sixth most common tumor and the third leading cause of cancer mortality globally. Hepatocellular carcinoma (HCC) accounts for 75% to 85% of all liver cancer cases (1). China has the greatest number of cases, contributing to more than half of HCC cases in the world (2). Because of its occult nature and invasiveness, more than half of HCC patients, upon initial presentation, have advanced disease that is not amenable to surgical resection and locoregional therapies (3). HCC with distant metastasis or progression despite locoregional therapy has a dismal prognosis, with a 5-year survival rate of merely 20.3% for metastatic HCC (4).

HCC is marked for aberrant oncoagniogenesis and is highly vascularized due to the activities of vascular endothelial growth factor receptors (VEGFR), fibroblast growth factors receptors (FGFR) and platelet-derived growth factor receptors (PDGFR). Antiangiogenic tyrosine kinase inhibitors (TKIs) sorafenib and lenvatinib were demonstrated to extend the overall survival (OS) of treatment-naïve advanced HCC patients in Phase 3 clinical studies, including the SHARP and REFLECT trials (5, 6), and have been approved as first-line treatment for unresectable HCC in China, the USA, and other countries. However, the efficacy of these molecular-targeted agents for unresectable HCC is rather modest and fatal adverse events (AEs) occurred in 2% HCC patients treated with lenvatinib and 1% HCC patients treated with sorafenib, highlighting the need for novel and more effective and safer molecular-targeted agents and new therapeutic strategies.

Apart from anomalous angiogenesis, immune evasion remains another hallmark of liver cancer (7). Immune checkpoint inhibitors (ICIs) targeting programmed cell death protein (PD)-1 such as nivolumab and pembrolizumab have been shown to extend the survival of previously treated patients with advanced HCC in Phase 2 trials and approved as second-line treatment of advanced HCC (8, 9). However, subsequent Phase 3 studies did not show superiority of anti-PD-1 monotherapy compared with standard of care for the first-line or second-line treatment of HCC (10, 11).

Vessel normalization by antiangiogenic therapy may change the tumor microenvironment and lead to enhanced transmigration of immune cells, suggesting synergic activities of antiangiogenic therapy and immune therapy (12, 13). The clinical benefits of combining antiangiogenic agents and ICIs have been reported for a variety of solid tumors, including HCC (14, 15). In the Phase 3 IMbraver150 study (16), atezolizumab, an anti-PD-ligand [L]1 antibody, plus anti-VEGF antibody bevacizumab significantly improved the median OS and progression-free survival (PFS) over sorafenib with an acceptable safety profile. Recently, the combination therapy has been approved in the USA, China and other countries for unresectable or metastatic HCC without prior systemic therapy.

Considering the distinctive properties between antiangiogenic monoclonal antibodies and TKIs (17), combination treatment with ICIs plus antiangiogenic TKIs could offer a potential first-line treatment for patients with advanced HCC. In the Phase 1b KEYNOTE-524 trial (18), pembrolizumab, an ICI, plus lenvatinib, an antiangiogenic TKI, showed promising antitumor activity with a tolerable safety profile for treatment-naïve unresectable HCC. The result encourages further exploration of additional antiangiogenic TKIs in combination with ICIs for patients with advanced HCC in the first-line setting.

Sintilimab, a selective IgG4 anti-PD-1 monoclonal antibody, binds to PD-1 receptor with high affinity and blocks its interaction with PD-L1 and PD-L2. Anlotinib, an antiangiogenic TKI that targets VEGFR, FGFR, PDGFR, and c-Kit, has demonstrated broad antitumor activities in several tumor types (19). In a Phase 1b study, sintilimab plus anlotinib exhibited encouraging efficacy, durability, and tolerability as first-line therapy in advanced non-small cell lung cancer (NSCLC) (20). We were interested in exploring the antitumor activities of sintilimab in combination with anlotinib as first-line treatment for Chinese advanced HCC patients given that the two drugs were developed in China and have been approved for other tumor types in the country (21, 22). Hence, we conducted this prospective study to investigate the safety and efficacy of sintilimab in combination with anlotinib in the first-line setting for unresectable or metastatic HCC and to preliminarily explore potential biomarkers of the combination treatment.

## Patients and Methods

### Study Design and Population

This study is an open-label, single-arm, phase 2 trial of sintilimab plus anlotinib with a safety run-in for patients with advanced HCC at Jiangsu Province Hospital (Nanjing, China). The key inclusion criteria were: 1) adult patients (between 18 and 70 years) with advanced histologically or cytologically-confirmed HCC; 2) at least one measurable lesion based on Response Evaluation Criteria in Solid Tumors (RECIST) v1.1; 3) Eastern Cooperative Oncology Group (ECOG) performance status (PS) score of 0 or 1; 4) Barcelona Clinic Liver Cancer stage B (not amenable or refractory to local therapy) or C categorization (unresectable or metastatic); 5) Child-Pugh class A or B (Child-Pugh score ≤7); 6) adequate organ function; 7) a predicted life expectancy of at least 12 weeks. Patients were excluded if they had previously received molecular targeted therapies including sorafenib, lenvatinib, anlotinib and apatinib, immunotherapy with any anti-PD-1/PD-L1 monoclonal antibody, or any other systemic treatment. The study was conducted in accordance with the Declaration of Helsinki, following approval by the ethics committee of Jiangsu Province Hospital. The trial is registered with clinicaltrials.gov (NCT04052152). All patients provided written informed consent before undergoing any study-specific procedures.

### Treatment

All participants received sintilimab 200 mg intravenously on day 1 and anlotinib 12 mg orally once daily on days 1 to 14 every 3 weeks. The first six participants, who were enrolled at a 7-day interval during the safety run-in, received two cycles of sintilimab plus anlotinib to assess dose-limiting toxicities (DLTs). Treatment was continued until disease progression, development of intolerable toxicities, death, withdrawal of consent, initiation of new antitumor therapy, or a maximum treatment duration of 34 cycles, whichever occurred first. Best supportive care was provided at the discretion of the investigators.

In the safety run-in, DLTs were recorded and graded according to the Common Toxicity Standards of the National Cancer Institute (NCI CTCAE) version 5.0 and defined as any grade 3 hematologic toxicity for more than 7 days, grade 4 hematologic toxicity or > grade 3 nonhematologic toxicity. If DLTs occurred in ≤ 2 patients during the safety run-in phase, additional patients were enrolled to initiate the expansion stage of the study.

Tumor response was assessed by investigators per RECIST v1.1 and modified RECIST (mRECIST) every two cycles. Complete (CR) and partial response (PR) had to be confirmed radiologically at least 4 weeks apart. Patients who withdrew due to intolerable toxicities were evaluated for efficacy at the time of withdrawal if no evaluation had been done in the preceding 4 weeks and thereafter followed up radiologically every 6 weeks until disease progression or initiation of other antitumor therapy. Patients with PD on initial radiological evaluation were allowed to continue treatment until PD again or intolerance if their clinical tumor-related symptoms were notably improved or stabilized and they were judged to be able to benefit from continuous treatment by investigators. AEs were assessed throughout the treatment period and for up to 30 days after the last dose per NCI CTCAE version 5.0. A safety follow-up was done within 30 days ( ± 3 days) after the last dose and a survival follow-up was done every 1 month after the safety follow-up.

### Endpoints

The primary endpoints included safety and ORR, which was defined as the proportion of patients who achieved investigators-confirmed CR or PR per RECIST v1.1. Secondary endpoints included disease control rate (DCR) (the proportion of patients who achieved CR, PR, or SD as their best overall response), duration of response (DoR) (the time from the first recorded CR or PR to disease progression or death), PFS (the time from the date of the first dose of the study medications to the date of radiographic disease progression or death of any cause, whichever occurred earlier), which were assessed by the investigators per RECIST v1.1 and mRECIST, and OS (the time from the date of the first dose of the study medications to the date of death of any cause).

### Biomarker Analysis

Blood samples were collected at baseline and at the end of each treatment cycle. The plasma levels of α-fetoprotein (AFP) and lactate dehydrogenase (LDH) were determined using an automatic chemical analyzer. The AFP variation rate (Δ AFP) was defined as the percentage of change between the baseline and the nadir within 9 weeks after treatment and calculated using the following formula (23):


$$\Delta \text{AFP}\left(\%\right)=\left[\left({\text{AFP}}_{baseline}-{\text{AFP}}_{post-treatment}\right)/{\text{AFP}}_{baseline}\right]\times 100\%$$

AFP response was defined as a Δ AFP (%) greater than 20%. Changes in LDH levels between the baseline and the nadir within 9 weeks after treatment were also calculated.

In addition, the proportions of circulating CD16^+^CD56^+^ NK cells were determined using a flow cytometer (detailed in **Supplementary Methods**) (24). Furthermore, the Controlling Nutritional Status (CONUT) score (25), which was calculated based on serum albumin levels, total lymphocyte count, and total cholesterol as previously depicted, was further used to assess immune-nutritional status of HCC patients. Scores are classified as normal (score 0–1), mild (score 2–4), moderate (score 5–8), or severe (score 9–12) malnutrition.

### Statistical Analysis

Sample size was not based on statistical power considerations. A sample size of approximately 20 patients (N=6 for DLT evaluation in the safety run-in phase and N=14 for the expansion phase) was planned for the study.

Continuous data were expressed as median with range. Categorical data were expressed in number and percentage. Statistical analyses were done with R version 3.4.1. The safety and efficacy analysis sets included all the patients from the DLT phase and the expansion phase who received at least 1 dose of the study drugs. ORR and DCR were calculated with corresponding two-sided 95% confidence intervals (CIs) using the Clopper-Pearson method. The Kaplan-Meier method was used for the analysis of PFS and OS. Two sample *t*-test (Welch’s *t*-test) was used to compare the proportions of CD16^+^CD56^+^ NK cells in HCC patients who achieved CR or PR and those who had SD or PD. Log rank test was used to compare the survival functions among different subgroups. The safety set was analyzed mainly using descriptive statistics. All tests were two-tailed with a level of significance set at α ≤0.05.

### Data Availability

The datasets used and/or analysed during the current study are available from the corresponding author upon reasonable request.

## Results

### Patients

**Figure 1:**
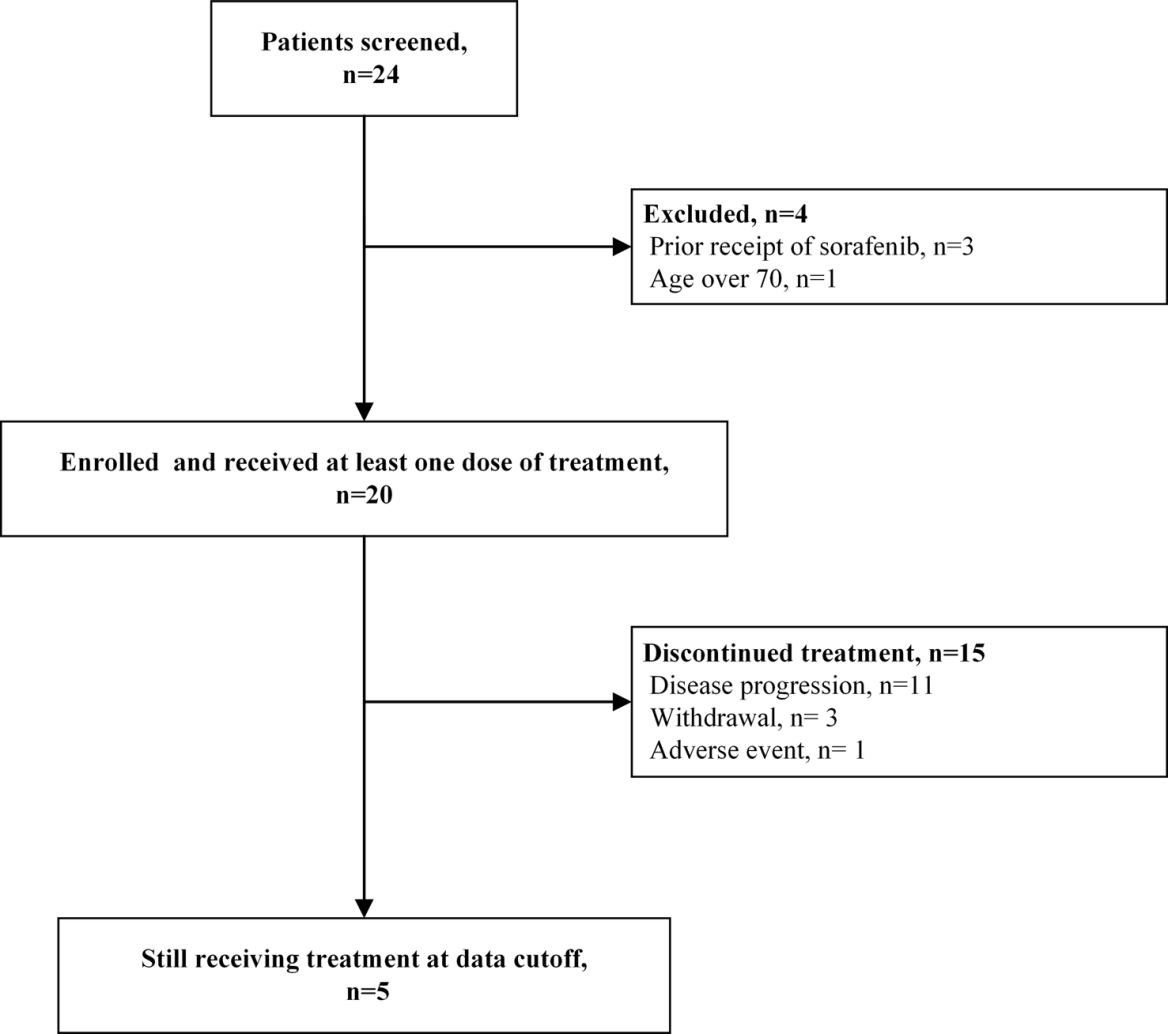
The study flowchart.

Between June 2019 and September 2020, 24 patients with unresectable or metastatic HCC were screened for eligibility. Four patients were excluded due to prior receipt of sorafenib (n=3) or inappropriate age (n=1). Finally, 20 patients were enrolled (shown **Figure 1**). Their median age was 56 years (range 41 to 70 years). Thirty percent (6/20) and 70% (14/20) of patients had ECOG PS of 0 and 1. Twenty-five percent (5/20) patients had BCLC stage B HCC and 75% (15/20) had BCLC stage C HCC. All patients had HBV infection. The liver function was Child–Pugh class A in 19 (95.0%) patients. Four (20.0%) patients had macrovascular invasion, and 14 (70.0%) had extrahepatic spread, with the most common extrahepatic sites being bones and lymph nodes (**Table 1**).

**Table 1:** Demographics and baseline characteristics patients.

Characteristics	All patients (N=20)
**Age, years**	56 (41 to 70)
**Sex**	
Male	18 (90.0)
Female	2 (10.0)
**Bodyweight**	
<60 kg	12 (60.0)
≥60 kg	8 (40.0)
**ECOG PS**	
0	6 (30.0)
1	14 (70.0)
**Child-Pugh classification**	
A	19 (95.0)
B_7_	1 (5.0)
**BCLC stage**	
B	5 (25.0)
C	15 (75.0)
**Infection**	
HBV^a^	20 (100.0)
HCV	0 (0.0)
**AFP**	
>400 ng/mL	7 (35.0)
≤400 ng/mL	13 (65.0)
**LDH**	
>271,UL	3 (15.0)
≤271,UL	17 (85.0)
**Alcohol consumption**	
Yes	6 (30.0)
No	14 (70.0)
**Macrovascular invasion**	
Yes	4 (20.0)
No	16 (80.0)
**Extrahepatic spread**	
Yes	14 (70.0)
No	6 (30.0)
**No. of involved disease sites per patient**	
1	6 (30.0)
2	9 (45.0)
3 or more	5 (25.0)
**Previous local regional therapy**	
Surgery	11 (55.0)
TACE	8 (40.0)
Local ablation Other	2 (10.0)

AFP, alpha-fetoprotein; ECOG, Eastern Cooperative Oncology; BCLC, Barcelona Clinic Liver Cancer; HBV, Hepatitis B virus; HCV, Hepatitis C virus; TACE, transarterial chemoembolization.

Data are median (range) or n(%).

a HBV infection is defined as HBsAg^+^ and HBV DNA <2, 000 IU/mL.

The study cutoff date was March 31, 2021.All patients in the study received at least 2 cycles of treatment with a median of 10 cycles (range 2 to 25 cycles) and a median duration of 7.3 months (range 1.1 to 21.6 months). Fifteen (75.0%) patients discontinued treatment due to PD (n=11, 55.0%), withdrawal (n=3, 15.0%) or AE (n=1, 5.0%). Five (25%) patients were still receiving treatment at the study cutoff date.

### Treatment Outcomes

No DLTs were reported in the 6 patients enrolled in the DLT phase of the study. Treatment-related AEs (TRAEs) of any grade occurred in all patients (100.0%) and the most common TRAEs (≥40%) were proteinuria (55.0%), hypoproteinemia (50.0%), decreased platelet count (50.0%) and hand-foot syndrome (40%) (**Table 2**). Grade 3 TRAEs were reported in 8 (40.0%) patients, the most common of which were decreased platelet count (10.0%) and increased γγ- glutamyl transferase (10.0%). No grade 4/5 TRAE was reported. Five (25%) patients developed immune-related AEs (irAEs). One patient had grade 2 immune pneumonitis, grade 3 hyperglycemia, and grade 3 bullous pemphigoid (dermatitis bullosa), while four patients had immune-related hypothyroidism (grade 2 in 3 patients and grade 1 in 1 patient).

**Table 2:** Treatment-related adverse events (TRAEs) in the safety set (N=20).

			n (%)	
Any TRAEs			20 (100.0)	
Grade 3 TRAEs			8 (40.0)	
Grade 4-5 TRAEs			0	
TRAEs leading to Sintilimab interruptions			2 (10.0)	
TRAEs leading to Anlotinib interruptions			6 (30.0)	
TRAEs leading to Anlotinib dose modification			12 (60.0)	
TRAEs leading to treatment discontinuation			1 (5.0)	
Most common TRAEs*	**n (%)**			
	**Any Grade**	**Grade1**	**Grade2**	**Grade3**
Proteinuria	11 (55.0)	8 (40.0)	2 (10.0)	1 (5.0)
Hypoproteinemia	10 (50.0)	10 (50.0)	0	0
Platelet count decreased	10 (50.0)	6 (30.0)	2 (10.0)	2 (10.0)
Hand-foot syndrome	8 (40.0)	5 (25.0)	2 (10.0)	1 (5.0)
AST increased	7 (35.0)	6 (30.0)	0	1 (5.0)
Hypertension	7 (35.0)	2 (10.0)	5 (25.0)	0
ALT increased	6 (30.0)	6 (30.0)	0	0
Neutrophil counts decreased	5 (25.0)	3 (15.0)	2 (10.0)	0
Leucocyte count decreased	5 (25.0)	2 (10.0)	3 (15.0)	0
Elevated bilirubin	4 (20.0)	2 (10.0)	2 (10.0)	0
Hyponatremia	4 (20.0)	4 (20.0)	0	0
Hypokalemia	4 (20.0)	3 (15.0)	0	1 (5.0)
Hypothyroidism	4 (20.0)	0	3 (15.0)	1 (5.0)
γ-GT increased	3 (15.0)	1 (5.0)	0	2 (10.0)
Arthrodynia	3 (15.0)	1 (5.0)	2 (10.0)	0
Sore throat	3 (15.0)	2 (10.0)	1 (5.0)	0
Bullous dermatitis	2 (10.0)	1 (5.0)	0	1 (5.0)

ALT, alanine transaminase; AST, aspartate transaminase; γ-GT, γ-glutamyl transferase.

*Listed are adverse events, as defined by the Common Toxicity Standards of the National Cancer Institute (version 5.0) that occurred in at least 10% of patients in the safety set.

TRAEs led to treatment interruption of sintilimab in 2 (10.0%) patients; TRAEs led to treatment interruption and dose modification of anlotinib in 6 (30.0%) and 12 (60.0%) patients, respectively. Discontinuation of both sintilimab and anlotinib occurred in one patient because of TRAEs.

**Figure 2:**
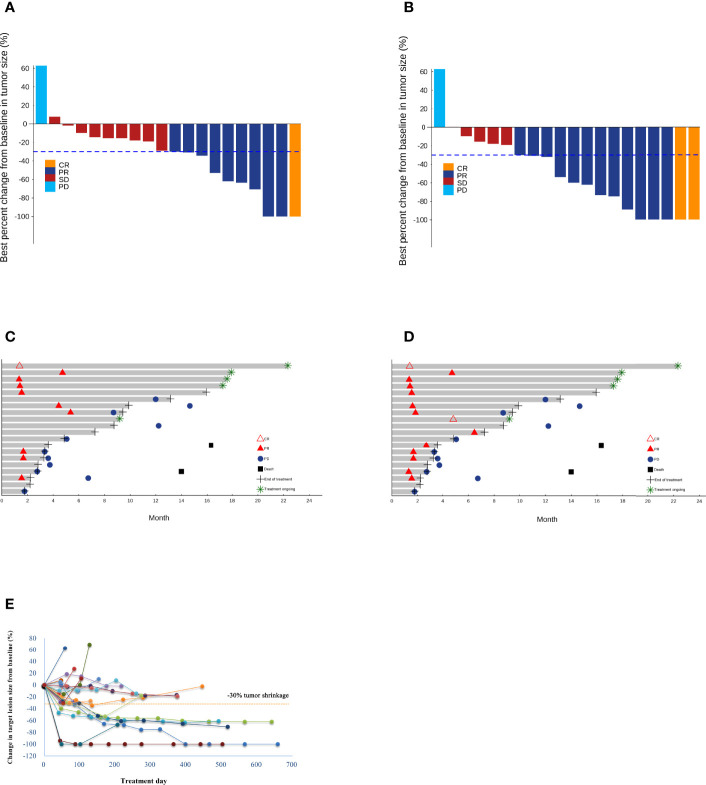
Waterfall plots of the best percentage changes for the sum of target lesion diameters after treatment are shown for individual patients with best objective response per RECIST v1.1 **(A)** and modified RECIST **(B)** as indicated by the color codes. Both investigator-confirmed and unconfirmed responses are included. The dotted line indicates a 30% reduction in the target lesion size. Swimmer plots of time to tumor response (months) of individual unresectable or metastatic hepatocellular carcinoma patients. Responses were evaluated by RECIST v1.1 **(C)** and modified RECIST **(D)**. Each bar represents one patient in the efficacy-evaluable population. CR, complete response; PD, progressive disease; PR, partial response; SD, stable disease. **(E)** The spider plot presents individual changes in tumor measurements over time relative to baseline tumor burden in the study patients. Patients are coded in different colors.

Regarding the primary endpoint, 1 patient achieved CR and 9 patients (including 3 unconfirmed cases) achieved PR per RECIST v1.1, with an ORR of 35.0% (95%CI 15.4% to 59.2%). Meanwhile, 2 patients achieved CR and 12 patients (including 3 unconfirmed cases) achieved PR per modified RECIST, with an ORR of 55.0% (95%CI 31.5% to 76.9%). In addition, 9 patients had SD per RECIST v1.1 and 5 patients had SD per modified RECIST, and the DCR was 95.0% (95%CI 73.1% to 99.9%) per both RECIST v1.1 and modified RECIST (**Table 3**) (shown in **Figures 2A, B**). The median time to treatment response was 1.6 months (95%CI 1.4 to 4.4 months) and the median DoR was 10.3 months [95%CI 1.7 to not reached (NR)] per RECIST v1.1. In addition, the median time to treatment response was 1.6 months (95%CI 1.4 to 2.7 months) and the median DoR was 13.1 months (95%CI 1.7 to NR) per modified RECIST (**Table 3**) (shown in **Figures 2C, D**). We further examined the relationship between changes in target lesion size over time relative to baseline tumor burden and duration of treatment. Patients with a greater than 30% reduction in target lesion size over time was associated with a longer duration of treatment compared to those with < 30% reduction in target lesions size (shown in **Figure 2E**).

**Table 3:** Summary of efficacy measures in the efficacy-evaluable population (N=20).

Outcomes	RECIST Version 1.1	Modified RECIST
**Best response^^^, n**		
CR	1	2
PR	9 (3 unconfirmed)	12 (3 unconfirmed)
SD	9	5
PD	1	1
**Objective response rate^#^, n (%)**	7 (35.0)	11 (55.0)
95% CI	15.4 to 59.2	31.5 to 76.9
Objective response rate^*^, n (%)	10 (50.0)	14 (70.0)
95% CI	27.2 to 72.8	45.7 to 88.1
Disease control rate^*^, n (%)	19 (95.0)	19 (95.0)
95% CI	73.1 to 99.9	73.1 to 99.9
Duration of response^*^, median, months	10.3	13.1
95% CI	1.7 to NR	1.7 to NR
Time to response^*^, median, months	1.6	1.6
95% CI	1.4 to 4.4	1.4 to 2.7
**PFS, median, months**	12.2	12.2
95% CI	3.8 to NR	3.8 to NR
**6-month PFS rate, %**	68.4	68.4
95% CI	50.4 to 92.9	50.4 to 92.9
**12-month PFS rate, %**	50.2	50.2
95% CI	31.4 to 80.1	31.4 to 80.1
**OS, median, months**	NR	NR
95% CI	16.3 to NR	16.3 to NR
**18-month OS rate, %**	77.9	77.9
95% CI	54.6 to 100	54.6 to 100

^Best response was evaluated by independent radiologic review per RECIST Version 1.1 and modified RECIST; ^#^Including confirmed responses only; *Including both confirmed and unconfirmed responses.

CR, complete response; PR, partial response; SD, stable disease; PD, progressive disease; PFS, progression-free survival; OS, overall survival; NR, not reached.

**Figure 3:**
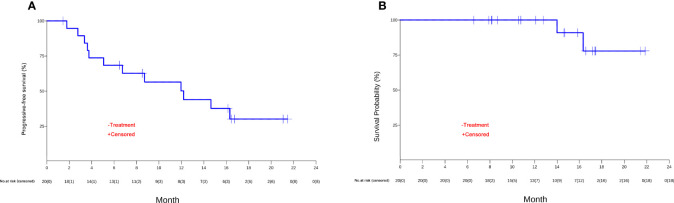
The Kaplan-Meier curves of **(A)** progression-free survival (PFS) and **(B)** overall survival (OS) of unresectable or metastatic hepatocellular carcinoma patients in the efficacy-evaluable population.

The patients were followed up for a median duration of 14.7 months (95% CI, 6.6 to 21.9 months). PFS events occurred in 12 (60%) patients. The median PFS was 12.2 months (95% CI, 3.8 to NR) (shown in **Figure 3A**), and the 12-month PFS rate was 50.2% (95% CI, 31.4% to 80.1%). OS events occurred in 2 (10%) patients. The median OS was not reached (95% CI, 16.3 months to NR) (**Figure 3B**), and the 18-month OS rate was 77.9% (95% CI, 54.6% to 100.0%) (**Table 3**).

### Biomarkers

**Figure 4:**
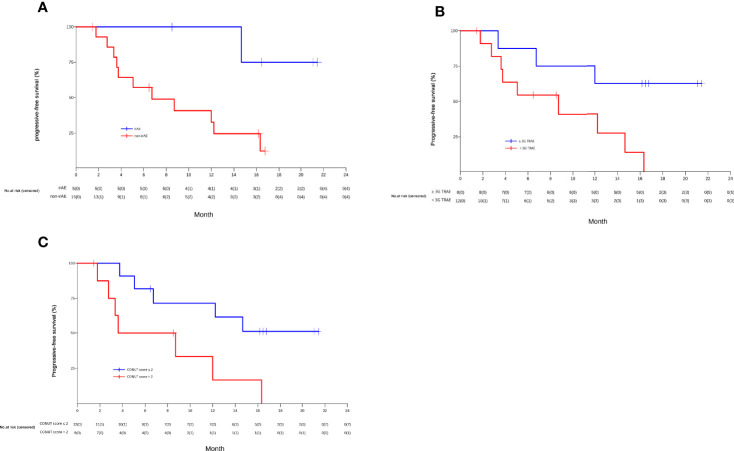
The Kaplan-Meier curves of PFS of unresectable or metastatic hepatocellular carcinoma patients in the efficacy-evaluable population stratified by the presence or absence of immune related-adverse events (irAEs) **(A)**, grade 3 treatment-related adverse events (TRAEs) **(B)**, and baseline controlling nutritional status (CONUT) score (> 2 vs. ≤2) **(C)**.

Patients who had an irAE had significantly longer median PFS than those who did not (NR, 95% CI 14.7 to NR vs. 6.8 months, 95% CI 3.8 to NR; *P*=0.02) (shown in **Figure 4A**). Furthermore, patients who had grade 3 TRAEs also had significantly longer median PFS than patients who did not (NR, 95% CI 12.0 to NR vs. 8.7 months, 95% CI 3.8 to NR; *P*=0.03) (shown in **Figure 4B**).

We further explored the relationship between changes in AFP levels over time relative to baseline AFP and duration of treatment. Overall, patients with a greater reduction in AFP levels over time tended to have a longer duration of treatment than those with a smaller reduction, no change, or an increase in AFP levels (shown in online **Supplementary Figure 1A**). Furthermore, patients with an AFP response exhibited an encouraging trend of greater PFS benefits than patients without an AFP response (16.3 vs. 3.6 months) (shown in online **Supplementary Figure 1B**). In addition, plasma LDH levels increased in 10 patients and declined in 10 patients within 8 weeks of treatment relative to baseline LDH. Patients with reduced LDH levels had a notably longer median PFS than those with increased LDH levels (NR, 95% CI, 8.7 to NR vs. 5.2months, 95% CI 3.4 to NR; *P*=0.020) (shown in online **Supplementary Figure 1C**).

We also examined the relationship between patient treatment response and the proportion of CD16^+^CD56^+^ NK cells at baseline. Patients who achieved CR or PR had a significantly higher median proportion of CD16^+^CD56^+^ NK cells than patients who had SD or PD (21.6% vs. 14.6%; *P*=0.026) (shown in online **Supplementary Figure 2**). In addition, the median CONUT score at baseline was 1.5 (range 0 to 8) (**Supplementary Table 1**). Twelve HCC patients had a CONUT score ≤ 2 and 8 had a CONUT score >2. Patients with a CONUT score ≤2 had a significantly longer median PFS than patients with a CONUT score >2 (NR, 95% CI 5.1 to NR vs. 6.2months, 95% CI 1.8 to NR; *P*=0.02) (shown in **Figure 4C**).

## Discussion

This single-arm phase II trial is the first study on the efficacy and safety of the combination regimen of sintilimab and anlotinib as first-line treatment for Chinese advanced HCC patients with HBV. Sintilimab and anlotinib achieved an ORR of 55.0% and a DCR of 95.0% per modified RECIST. Remarkably, the median PFS reached 12.2 months per both RECIST v1.1 and modified RECIST. Grade 3 TRAEs occurred in 40% of the patients and were manageable; no grade 4/5 TRAEs were reported. These findings together suggest that the combination of ICI sintilimab and antiangiogenic TKI anlotinib is safe and provides promising clinical benefits for unresectable or metastatic HCC patients who have received no prior systemic therapy.

Overall, sintilimab plus anlotinib had an acceptable safety profile. No new TRAE of concern was reported in the study patients. Grade 3 TRAEs occurred in 8 (40.0%) patients, which is comparable to that (36.0%) in the IMbrave150 trial (16), but apparently lower than that (74.7%) with camrelizumab, an anti–PD-1 monoclonal antibody, plus apatinib, a VEGFR2 inhibitor, as first-line treatment for advanced HCC in the phase II RESCUE trial (26). Five (25.0%) patients had an irAE. Due to combination therapy, it is hard to discern an irAE and the number of irAEs in the current trial might be higher than reported. Of note, it has been suggested that the occurrence of irAEs could herald treatment response to ICI therapy (27, 28). Indeed, we found that patients who had an irAE had significantly longer median PFS than patients who did not. We also observed a significantly longer median PFS in patients who had grade 3 TRAEs than patients who did not. It is worthy to further validate the findings and explore the biological mechanisms underlying the association between the occurrence of irAEs and treatment efficacy.

Sintilimab plus anlotinib had an ORR of 35.0% per RECIST v1.1, which is consistent with other combination regimens of an ICI and an antiangiogenic TKI such as in the KEYNOTE-524 trial (18), the RESCUE trial (26), and the AK105-203 trial (29), which achieved an ORR between 31% and 36%. Meanwhile, sintilimab plus bevacizumab in the ORIENT-32 study had an ORR of 20.0% and atezolizumab plus bevacizumab yielded an ORR of 27.3% in the milestone IMbrave150 study (16, 30), suggesting that small molecule inhibitors of oncoangiogenesis might be more effective than antiangiogenic monoclonal antibodies in enhancing patient treatment response when in combination with an ICI. Although cross-study comparisons have many limitations, the longest median PFS and DCR reported in studies of first-line combination of an ICI and an antiangiogenic agent for patients with advanced HCC are less than 12 months and 90%, respectively. In this study, sintilimab plus anlotinib achieved a median PFS of 12.2 months and a DCR of 95%, which are very encouraging. Currently, several trials involving various combinations of immunotherapeutic agents plus antiangiogenic molecules are ongoing, the efficacy of these various combinations may depend on subtle differences in the antiangiogenic agents and the results are eagerly awaited.

There is lack of an effective biomarker for the efficacy of ICI therapy of HCC (31, 32). In this study, we evaluated the value of several routinely collected variables as biomarkers of response to ICIs. Notably, the changes of plasma AFP and LDH levels, the proportion of CD16^+^CD56^+^ NK cells and CONUT scores at baseline tended to be associated with the treatment outcomes of advanced HCC. Early AFP response has been shown to be a useful surrogate biomarker of response to antiangiogenic therapy and ICIs for advanced HCC (33–35). The current study further showed that greater decline in post-treatment AFP levels was associated with longer duration of treatment, and AFP responders exhibited a remarkable trend of greater PFS benefits (16.3 months vs. AFP non-responders 3.6 months), suggesting that AFP response could be a potential biomarker of response to combination therapy of an anti-angiogenesis agent and an ICI. LDH, an indirect marker of hypoxia, has been shown to be an adverse predictor of response to ICIs in a variety of cancer types (36). However, its role remains undefined as a predictor of response to combination therapy for advanced HCC with an anti-angiogenesis agent and an ICI. In the current study, advanced HCC patients with lower post-treatment LDH levels had significantly longer median PFS, suggesting a possible role of LDH levels in stratifying advanced HCC patients for combined anti-angiogenesis and ICI therapy. In addition, NK cells play an important role in innate defense against HCC (37). Small molecule multikinase inhibitor sorafenib was reported to exert anti-HCC activities by modulating the ratio of peripheral CD56^bright^CD16^-^ and CD56^dim^CD16^+^ NK cells (38). Similarly, this study showed that a higher proportion of CD16^+^CD56^+^ NK cells at baseline was associated with better treatment response (CR or PR). Meanwhile, no association was found between the proportion of CD3^+^ cells, CD3^+^CD4^+^ cells, CD3^+^CD8^+^ cells, CD19^+^ cells, CD4^+^/CD8^+^ cells, or Treg cells and treatment response (Data not shown). The CONUT index, an immunonutritive scoring system, was shown to be an important prognostic predictor of HCC patients treated with multikinase inhibitor Lenvatinib (39). In this study, we found that patients with a CONUT score ≤2 had a significantly longer median PFS than patients with a CONUT score > 2, further suggesting that CONUT score may provide an early clinically meaningful and convenient biomarker for stratifying advanced HCC patients for combination therapy with an anti-angiogenesis agent and an ICI. Of note, the current study provides the first piece of evidence that CONUT score is associated with the therapeutic outcome of HCC patients treated with immunotherapy. As the sample size of the current study is rather limited, the predictive role of these biomarkers individually or in combination should be further explored in future prospective studies involving larger sample sizes.

The major limitation of this study is that this is a single arm and single institution study with a small sample size. It is also worth noting that the study was conducted during the ongoing COVID-19 pandemic, which impacted on patient enrollment and treatment.

In conclusion, the current trial has shown that sintilimab plus anlotinib improve the treatment response and extend the survival of unresectable or metastatic HCC patients who have received no prior systemic therapy, with a good safety profile and manageable toxicities. The findings further confirmed the effectiveness of a combination of a PD-1 inhibitor with an antiangiogenic TKI for advanced HCC patients in the first line setting and sintilimab plus anlotinib are worthy of being further investigated in randomized controlled trials with a larger population.

## Data Availability Statement

The raw data supporting the conclusions of this article will be made available by the authors, without undue reservation.

## Ethics Statement

The studies involving human participants were reviewed and approved by the ethics committee of Jiangsu Province Hospital. The patients/participants provided their written informed consent to participate in this study.

## Author Contributions

Conceptualization: XC, YS, and XCL; data curation: XC, WL, XW, and FZ; formal analysis: XC, WL, and XW; funding acquisition: XC and YS; investigation: XC, WL, XW, FZ, DW, HW, YG, XL, XQ, JH, CL, YX, JR, XD, QS, JT, XCL, and YS; project administration: YS; roles/writing - original draft: XC, WL, and JT; writing - review and editing: XC, WL, XW, FZ, DW, HW, YG, XL, XQ, JH, CL, YX, JR, XD, QS, JT, XCL, and YS. All authors contributed to the article and approved the submitted version.

## Funding

The work was supported by Jiangsu Province 333 High Level Talents Project (XC), Innovation Funds from Chinese Society of Clinical Oncology Youth Committee (Y-young2019-060 to XC), the Advanced Health Talent of Six-One Project of Jiangsu Province (LGY2017069 to XC), the Joint Research Project by Southeast University and Nanjing Medical University (3207027381 to XC), and Pukou District Social Cause Science and Technology Development Project in 2020 (number S2020-21).

## Conflict of Interest

The authors declare that the research was conducted in the absence of any commercial or financial relationships that could be construed as a potential conflict of interest.

## Publisher’s Note

All claims expressed in this article are solely those of the authors and do not necessarily represent those of their affiliated organizations, or those of the publisher, the editors and the reviewers. Any product that may be evaluated in this article, or claim that may be made by its manufacturer, is not guaranteed or endorsed by the publisher.

## Supplementary Material

Supplementary Figure 1 **(A)** The spider plot presents individual changes in AFP levels over time relative to baseline AFP in the study patients. Patients are coded in different colors. **(B)** The Kaplan-Meier curves of progression-free survival (PFS) of efficacy-evaluable patients stratified by the presence or absence of AFP response. **(C)** The Kaplan-Meier curves of PFS of efficacy-evaluable patients stratified by LDH changes between the baseline and the nadir within 9 weeks after treatment.

Supplementary Figure 2 Box and whisker graphs show the proportion of CD16^+^CD56^+^ NK cells in the plasma at baseline of HCC patients who achieved CR or PR (left) or who had SD or PD (right). Boxes represent the 25^th^ to 75^th^ percentiles, and whiskers extend to the highest and lowest values within 1.5× the upper/lower quartile distance, with outliers shown as dots.
