# Supplementary figures and images for: Safety and Efficacy of Sintilimab and Anlotinib as First Line Treatment for Advanced Hepatocellular Carcinoma (KEEP-G04): A Single-Arm Phase 2 Study
Source: Front Oncol. 2022 May 31;12:909035. doi: 10.3389/fonc.2022.909035 (PMC9197581; doi:10.3389/fonc.2022.909035)

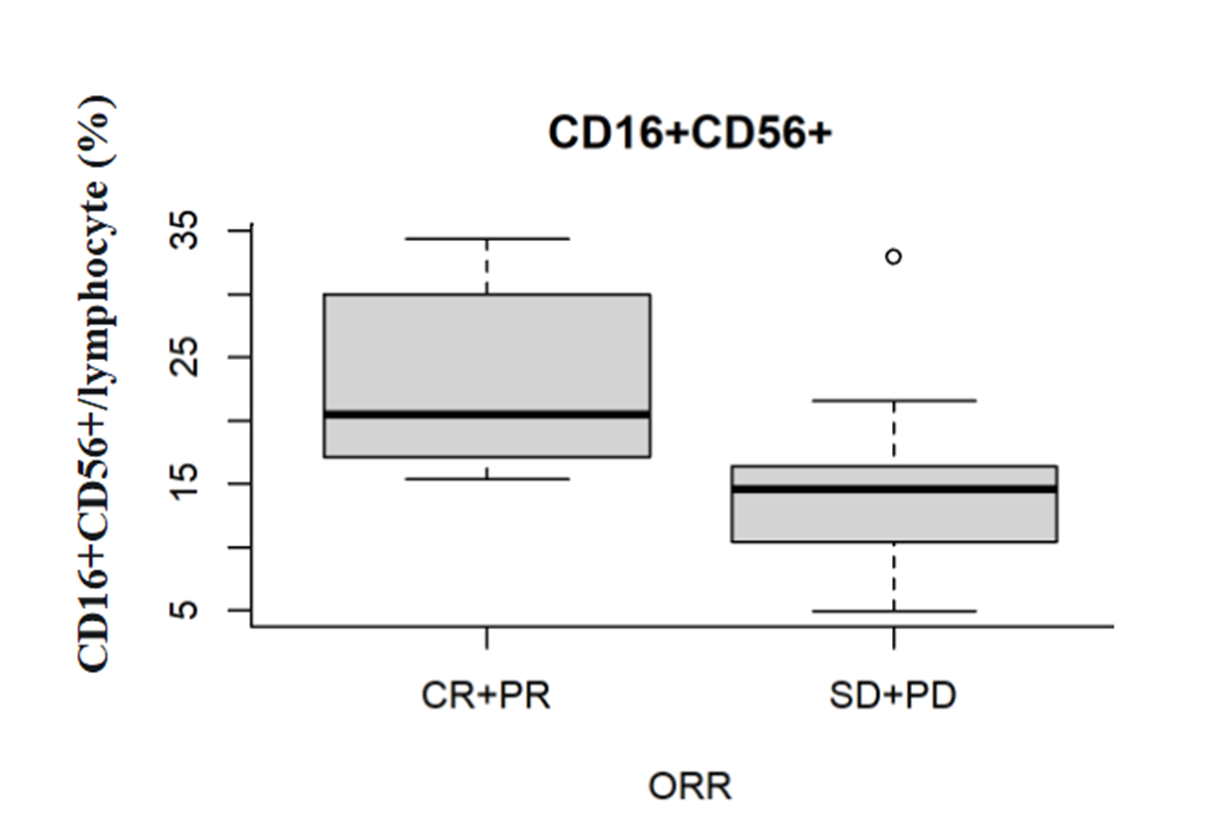

Supplement: Supplementary Figure 1 — (A) The spider plot presents individual changes in AFP levels over time relative to baseline AFP in the study patients. Patients are coded in different colors. (B) The Kaplan-Meier curves of progression-free survival (PFS) of efficacy-evaluable patients stratified by the presence or absence of AFP response. (C) The Kaplan-Meier curves of PFS of efficacy-evaluable patients stratified by LDH changes between the baseline and the nadir within 9 weeks after treatment. [file Image_1.tif]

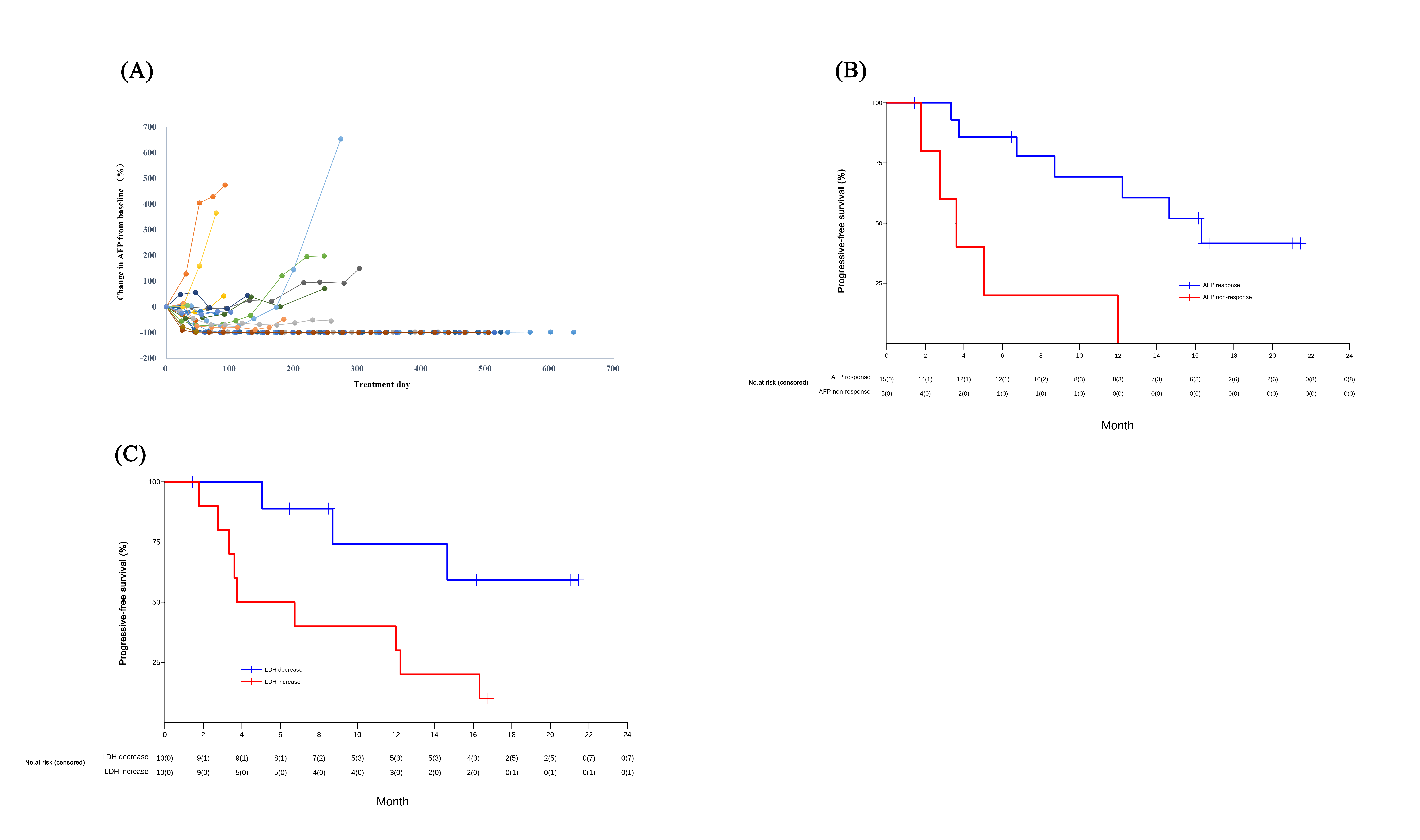

Supplement: Supplementary Figure 2 — Box and whisker graphs show the proportion of CD16+CD56+ NK cells in the plasma at baseline of HCC patients who achieved CR or PR (left) or who had SD or PD (right). Boxes represent the 25th to 75th percentiles, and whiskers extend to the highest and lowest values within 1.5× the upper/lower quartile distance, with outliers shown as dots. [file Image_2.tif]
