# Supplementary material for: Safety and Efficacy of Sintilimab and Anlotinib as First Line Treatment for Advanced Hepatocellular Carcinoma (KEEP-G04): A Single-Arm Phase 2 Study
Source: Front Oncol. 2022 May 31;12:909035. doi: 10.3389/fonc.2022.909035 (PMC9197581; doi:10.3389/fonc.2022.909035)
Supplement: Supplementary file 3 [file DataSheet_1.docx]

**Supplementary Methods**

*Analysis of circulating CD16+CD56+ NK cells*

The proportions of circulating CD16^+^CD56^+^ NK cells were determined using a flow cytometer. Briefly, peripheral venous blood was collected and anticoagulated with EDTA from each patient in a tube and kept at room temperature. One hundred μL of freshly collected blood was transferred into a flow-specific tube and then 20 μL of 4-color TBNK reagent (Ref # 6607013 including CD45-FITC, CD4-PE, CD8-ECD, CD3-PC5 and Ref # 6607073 including CD45-FITC, CD56-PE, CD19-ECD, CD3-PC5, Beckman Coulter, USA) was added per manufacturer’s instruction. After incubation at room temperature for 30 min in the dark, the mixture was incubated with 2.5 mL red blood cell lysis buffer (Sigma-Aldrich) for 15 min at room temperature in the dark. Then the sample was analyzed using a CYTOMICS-FC500 flow cytometer（Beckman Coulter）with CXP software.

**Supplementary Figure Legends**

**Supplementary Figure 1.** (**a**) The spider plot presents individual changes in AFP levels over time relative to baseline AFP in the study patients. Patients are coded in different colors. (**b**) The Kaplan-Meier curves of progression-free survival (PFS) of efficacy-evaluable patients stratified by the presence or absence of AFP response. (**c**) The Kaplan-Meier curves of PFS of efficacy-evaluable patients stratified by LDH changes between the baseline and the nadir within 9 weeks after treatment.

**Supplementary Figure 2.** Box and whisker graphs show the proportion of CD16^+^CD56^+^ NK cells in the plasma at baseline of HCC patients who achieved CR or PR (left) or who had SD or PD (right). Boxes represent the 25^th^ to 75^th^ percentiles, and whiskers extend to the highest and lowest values within 1.5× the upper/lower quartile distance, with outliers shown as dots.
