# Supplementary material for: Safety and Efficacy of Sintilimab and Anlotinib as First Line Treatment for Advanced Hepatocellular Carcinoma (KEEP-G04): A Single-Arm Phase 2 Study
Source: Front Oncol. 2022 May 31;12:909035. doi: 10.3389/fonc.2022.909035 (PMC9197581; doi:10.3389/fonc.2022.909035)
Supplement: Supplementary file 4 [file Table_1.docx]

**Supplementary Table 1. Controlling Nutritional Status index score: assessment of malnutritional state**

| **Parameter** | **Malnutritional state** | | | |
| --- | --- | --- | --- | --- |
|  | **Normal** | **Mild** | **Moderate** | **Severe** |
| **Albumin (g/dl)** | **≥3.50** | **3.00-3.49** | **2.50-2.99** | **<2.50** |
| **Score** | **0** | **2** | **4** | **6** |
| **Total lymphocyte count (mg/ml)** | **≥1600** | **1200-1599** | **800-1199** | **<800** |
| **Score** | **0** | **1** | **2** | **3** |
| **Total cholesterol (mg/dl)** | **≥180** | **140-179** | **100-139** | **<100** |
| **Score** | **0** | **1** | **2** | **3** |
| **Total score** | **0-1** | **2-4** | **5-8** | **9-12** |
